# Quantification of Nitric Oxide Concentration Using Single-Walled Carbon Nanotube Sensors

**DOI:** 10.3390/nano11010243

**Published:** 2021-01-18

**Authors:** Jakob Meier, Joseph Stapleton, Eric Hofferber, Abigail Haworth, Stephen Kachman, Nicole M. Iverson

**Affiliations:** 1 Department of Biological Systems Engineering, University of Nebraska-Lincoln, Lincoln, NE 68583, USA; jakob.meier@huskers.unl.edu (J.M.); joseph.stapleton@huskers.unl.edu (J.S.); eric.hofferber@huskers.unl.edu (E.H.); ahaworth1of6@huskers.unl.edu (A.H.); 2 Department of Statistics, University of Nebraska-Lincoln, Lincoln, NE 68583, USA; steve.kachman@unl.edu

**Keywords:** nitric oxide, concentration quantification, reactive species, carbon nanotube sensors, spatial detection, temporal detection

## Abstract

Nitric oxide (NO), a free radical present in biological systems, can have many detrimental effects on the body, from inflammation to cancer. Due to NO’s short half-life, detection and quantification is difficult. The inability to quantify NO has hindered researchers’ understanding of its impact in healthy and diseased conditions. Single-walled carbon nanotubes (SWNTs), when wrapped in a specific single-stranded DNA chain, becomes selective to NO, creating a fluorescence sensor. Unfortunately, the correlation between NO concentration and the SWNT’s fluorescence intensity has been difficult to determine due to an inability to immobilize the sensor without altering its properties. Through the use of a recently developed sensor platform, systematic studies can now be conducted to determine the correlation between SWNT fluorescence and NO concentration. This paper explains the methods used to determine the equations that can be used to convert SWNT fluorescence into NO concentration. Through the use of the equations developed in this paper, an easy method for NO quantification is provided. The methods outlined in this paper will also enable researchers to develop equations to determine the concentration of other reactive species through the use of SWNT sensors.

## 1. Introduction

Nitric oxide (NO), a naturally occurring chemical in the body, plays a role in the vascular, immune, and central nervous system [1,2,3,4,5,6,7]. NO has also been implicated in the progression of several diseases, such as cancer, sepsis, multiple sclerosis, and various autoimmune diseases [1,2,3,4,5,6,7,8,9,10,11,12,13,14,15,16,17,18,19,20,21,22,23,24]. Unfortunately, NO has been reported to have both positive and negative effects on disease progression [8,9,10,11,12,13,14,15,16,17,18,19,20,21].

The contradiction in NO’s role in disease progression is likely due to the difficulty of accurate and rapid detection of NO, which is due to NO’s low concentration, ranging from 1 nM to 1 µM, and short half-life, estimated at less than 1 ms to multiple seconds in biological samples [2,25,26,27,28,29,30,31,32,33,34,35,36,37,38,39].

Popular methods of NO detection, including the Griess assay, horseradish peroxidase (HRP), and various electrochemical probes, suffer from limitations, including detection of upstream or downstream products of NO, rather than NO itself, and a lack of spatial detection (Table 1) [40,41,42]. Downstream measurements of NO are frequently inaccurate because NO decays into different molecules, such as sodium formate [43], methemoglobin [27], or nitrogen dioxide [44], depending on the chemical makeup of the environment. Upstream assays for NO encounter similar quantification issues, since the formation of NO is dependent on multiple cell-specific characteristics [41]. The conflicting reports about NO’s concentration demonstrate the need for a quantification method capable of directly detecting biologically relevant NO concentrations.

Single-walled carbon nanotubes (SWNT) emit light in the near infrared (nIR) range when excited, and when wrapped with specific polymers, they become optical sensors for a wide array of analytes, including NO, reactive oxygen species, insulin, and dopamine [45,46,47,48,49,50,51,52]. SWNT sensors react to their analyte of interest with an increase or decrease in fluorescence intensity and/or a blue or red shift in their wavelength [45,49,53,54]. Researchers are interested in developing SWNT as sensors for biological applications since their emission wavelength falls within the near-infrared range, an area in which water and blood have limited interference, and they do not photobleach, therefore providing a long-term fluorescence sensor [55,56,57].

When a (6, 5) SWNT is wrapped with single-stranded (AT)_15_, a 30-m strand of DNA, a fluorescence quenching NO sensor is created [45]. The (AT)_15_-wrapped SWNT maintains a constant fluorescence intensity until it is exposed to NO; once NO is introduced to the SWNT, the fluorescence intensity will decrease [45]. This decrease in fluorescence intensity does not occur when similar reactive oxygen and nitrogen species are exposed to the SWNT, it only occurs when exposed to NO [45]. Due to their lack of photobleaching, real-time response rate, analyte specificity, and ability to detect NO, not a precursor or downstream product of NO, these SWNT sensors have many unique properties that cannot be found in other NO sensors [45,57,58,59,60,61].

**Table 1:** Comparison of three of the frequently used nitric oxide (NO) sensors to the (AT)_15_ single-walled carbon nanotube (SWNT) sensor. ** The range of detection (µM) for the SWNT has not previously been determined and will be shown in this paper.

Name	Range of Detection (µM)	Analyte Measured	Real-Time Sensing	Spatial Resolution
Griess [62]	250–200	Nitrite	No	No
Electrochemical probe	0.1–3000 [42] 0.01–0.3 [63]	NO	Yes	No
Horseradish peroxidase [64]	0.085–1.3	NO	No	No
SWNT sensors [45]	**	NO	Yes	Yes

Unfortunately, the (AT)_15_ SWNT sensor does not have a linear fluorescence quenching rate compared to NO concentration, so the determination of the actual NO concentration, as opposed to quantification of the changes in the concentration, has never before been determined. In this paper, we demonstrate the success of our research aim, which was to develop a mathematical model that converts the change in SWNT fluorescence into NO concentration.

## 2. Materials and Methods

### 2.1. SWNT Sensors

SWNT sensors were made as previously described [53]. Briefly, single-stranded (AT)_15_ DNA was added to (6, 5) SWNT in nanopure water in a 2:1 ratio (Integrated DNA Technologies, Coralville, IA, USA and Sigma-Aldrich, St. Louis, MO, USA). The SWNT and DNA solution was placed in a bath sonicator for 10 min, tip sonicator for two 20 min periods, and then centrifuged twice (ThermoFisher, Waltham, MA, USA and Qsonica, Newtown, CT, USA). The remaining supernatant was then analyzed on an ultraviolet–visible spectrometer (UV–Vis) (Beckman Coulter, Brea, CA, USA) to determine its concentration [45].

### 2.2. Attachment of SWNT to Glass Surface

The SWNT sensors were adhered to a glass slide using a previously described method [65]. Briefly, the glass slides were treated over the course of five days with piranha solution, 3-glycidyloxypropyl trimethoxy-silane (GPTMS), and avidin, before incubating with biotinylated SWNT (Sigma-Aldrich, St. Louis, MO, USA and Integrated DNA Technologies, Coralville, IA, USA) [65].

### 2.3. Nitric Oxide Solution

Both a NO and NO-free control solution were made as previously described [66]. Briefly, 12 mL of saline was placed in two sealed round-bottomed flasks. Argon was bubbled into both flasks for 20 min to de-oxygenate the saline, then NO was bubbled through a single flask for 5 min to create an NO solution (Matheson Tri-Gas, Irving, TX, USA).

### 2.4. NO Concentration Quantification via Horseradish Peroxidase

NO concentration was determined as previously described by Qiang et al. [64]. Briefly, NO was mixed with a horseradish peroxidase solution (final concentration 1.36 µM) (ThermoFisher, Waltham, MA, USA). The absorbance values at 405 and 420 nm were collected and used to calculate NO concentration via Qiang et al.’s formula [64].

### 2.5. Preparation of Slides for Imaging

Before imaging, the slides were tightly fitted to a holder by means of thermal expansion. They were then allowed to cool and reach thermal equilibrium, before adding 4 mL of saline. The slides were placed on the microscope and imaged for 2 min to establish a baseline. After that, 400 µL of saline was withdrawn from the slide holder, to ensure the slide stayed in focus when the 400 µL of NO was injected.

### 2.6. Detecting SWNT Response to NO (Fluorescence Measurements)

Sensitivity and reactivity of the SWNT sensors to NO was determined using the custom-built, hyperspectral near infrared microscope (Photon, etc., Montreal, QC, Canada). The microscope excites samples with a 2 W laser (561 nm), collects the emission signal with a volume Bragg grating to choose the specific wavelength of interest, and records the data with an InGaAs camera (Xenics, Beverly, MA, USA). SWNT fluorescence was monitored while solutions of NO at different concentrations were added (400 µL of NO solution to a 3600 µL saline bath). Images were collected every 200 milliseconds for 6.5 min, with the NO injection at the 2.5 min timepoint. A custom developed MATLAB program (Supplementary File 1) (MathWorks, Natick, MA, USA) was used to quantify h5 files (a file type specific to our imaging system).

### 2.7. Mathematical Analysis

Mathematical analysis was performed under the supervision of a trained statistician. First, the average brightness for each frame of the video was extracted via a custom-developed program (Supplementary File 1) and smoothed using a standard three-point median filter.

The signal intensity difference was found by averaging the last quarter of the data collected before injection for the initial value, and the final quarter of the data collected after the injection of NO for the final value, and then subtracting the final value from the initial value.

The slope was determined using the local maximum before the injection of NO and local minimum in the first quarter of the data after the injection of NO.

Each collection of NO concentrations was averaged, and the linear section of the graph was fit with an equation correlating NO concentration and either fluorescent signal intensity difference or the slope of the fluorescent signal.

## 3. Results and Discussion

An important aspect for analyzing the change in SWNT fluorescence due to NO exposure is the stabilization of the SWNT. Therefore, it was important that the sensor-coated slides be analyzed within a device that kept them from moving and also allowed for the saline bath used in the experiments. A slide holder was 3D printed to fulfill this purpose. Before use, the slide holders were expanded via heat, and then the slide was placed in the holder, which was allowed to cool, creating a tight seal between the slide and the holder.

**Figure 1:**
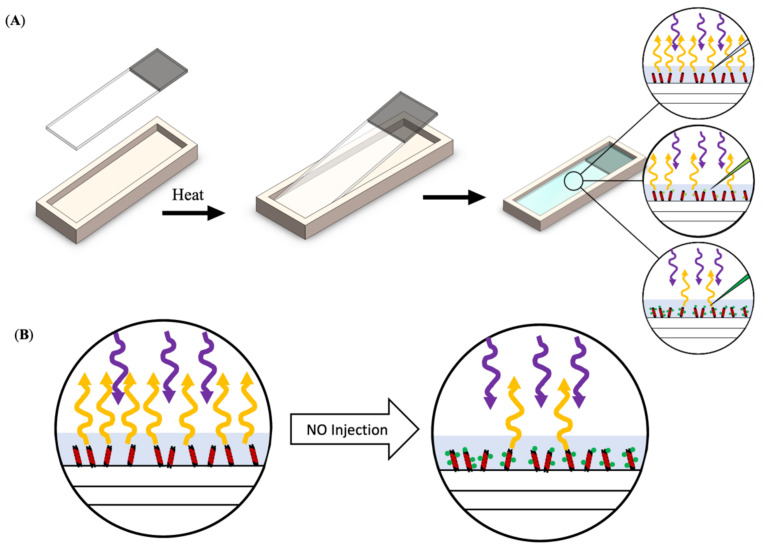
Schematic of testing process. (**A**) Sensor-coated slide was placed into a heated slide holder and then bathed in saline (25 °C). The slide/slide holder was placed on the upright microscope and imaged before and after the addition of various NO concentrations. With the addition of increasing concentrations of NO, there is a decrease in the fluorescent signal emitted by the SWNT. (**B**) A schematic showing the change in fluorescence emission with the addition of NO.

Once the sensor-coated slide was stabilized it was imaged with a custom-made upright microscope. The SWNT were excited via a 561 nm wavelength laser, and the subsequent emissions at 990 nm were read by a 20× objective with an exposure time of 200 ms for a total duration of 6.5 min. The sensors were exposed to various concentrations of NO as well as a non-NO control while fluorescence intensity readings were collected (Figure 1). Unfortunately, the ratio of the SWNT to NO cannot be determined, since SWNT is measured by fluorescence intensity, not by number, but the number of SWNTs on the surface of the slides remained constant, so the change in the NO concentration led to a change in the SWNT to NO ratio.

**Figure 2:**
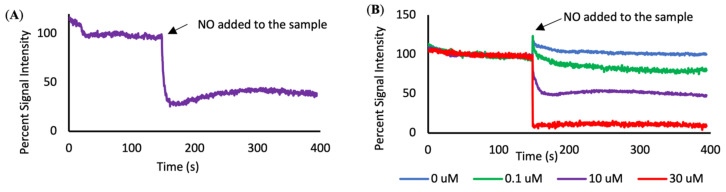
Fluorescence quenching curves. (**A**) An example quenching curve before and after the addition of 10 µM NO. (**B**) Signal intensity over time, forming quenching curves that display the average response of the SWNT sensors to different concentrations of NO.

**Figure 3:**
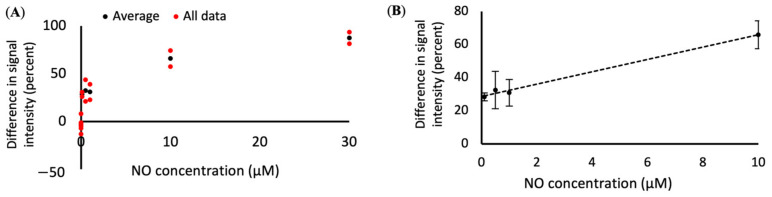
Difference in signal intensity for different NO concentrations. (**A**) The change in fluorescence intensity of the SWNT compared to NO concentration, with individual data points in red and averaged data points in black and (**B**) concentration curve (*x* = (*y* − 28.59)/3.73, with *x* = NO concentration in µM and *y* = difference in fluorescence intensity) that can be used to convert SWNT fluorescence changes into NO concentration. *R*^2^ value = 0.99.

The SWNT sensors respond to the different concentrations of NO by quenching to different extents (Figure 2). An addition of 0.1 µM or higher concentrations of NO resulted in a measurable decrease in fluorescence when compared to the non-NO control (*n* = 3–7). The addition of higher concentrations of NO resulted in a lower final fluorescence intensity when compared to the final intensity of samples exposed to lower NO concentrations (Figure 3A).

The lower limit of detection for the SWNT was found to be 0.1 µM, with concentrations of NO below 0.1 µm resulting in changes of fluorescence that were within the noise range of the 0 µm control samples. With the current system, the SWNT does not have a discernable upper limit for detection, but it does have an upper limit for differentiation between concentrations. When 30 µM NO is added to the system, the SWNT becomes fully saturated. Increasing the NO concentration beyond that point will not change the observable fluorescence intensity. Therefore, we have set the functional upper limit of quantification to be 30 µM.

The goal of this project was to develop a mathematical model that correlates the response of the SWNT sensors to NO concentration. While the SWNT respond to NO over a wide variety of concentrations, the response is not always linear. However, the statistical analysis of the data, as described in the materials section, was limited to a linear section of the graph (Figure 3B), to ensure a more accurate curve fit. The quenching was found to have a linear fit within the range of 0.1 to 10 µM when determined through analysis of the difference in initial vs. final signal intensity. The equation comparing the drop in fluorescence intensity to NO concentration is *x* = (*y* − 28.59)/3.73, where y is the change in fluorescence and *x* is the concentration of NO in µM.

**Figure 4:**
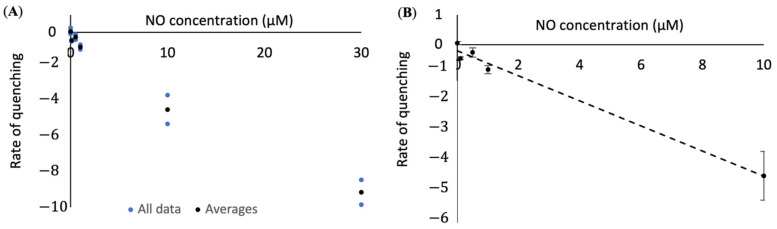
Slope of signal intensity after the addition of different concentrations of NO. (**A**) The slope of the fluorescence intensity of the SWNT compared to NO concentration, with individual data points in blue and averaged data points in black and (**B**) concentration curve (*x* = −(*y* + 0.21)/0.42, with *x* = NO concentration in µM and *y* = slope of signal intensity) that can be used to convert SWNT fluorescence changes into NO concentration. *R*^2^ value = 0.99.

Since the lower concentrations of NO are of interest in biological settings, we attempted to create an analysis method that is accurate at lower concentrations of NO. We found that by comparing the slope of the fluorescent signal with NO concentration we were able to get a much better fit for our data at low concentrations, creating a sensor with a range of 0 to 10 µM (Figure 4). The equation comparing the slope of the fluorescence intensity to NO concentration is *x* = −(*y* + 0.21)/0.42, where y is the slope of the fluorescent signal and *x* is the concentration of NO in µM.

We are choosing to report both methods of NO concentration quantification since there are situations for which each method is preferable. When a researcher is interested in the total concentration of NO added to a system, the change in SWNT’s initial to final fluorescence will provide the necessary information without the complication of noise in the system as more/less NO is being released in short time spans. Whereas the quantification of NO concentration via the slope of the fluorescent signal will be beneficial for situations in which temporal data or information about low concentrations of NO is required.

These models do have some limitations, including the fact that the SWNT must be adhered to a glass slide, meaning that extracellular NO can be quantified, but intracellular NO concentrations are not currently quantifiable. The results also take more time to obtain and process when compared to a traditional electrochemical probe. However, with the development of this model, NO concentrations can be analyzed spatially down to the µm scale, which is not feasible with current electrochemical probe technology. Our SWNT sensing system allows for repeated quantification of NO in both a spatial and temporal fashion, which is a feat that none of the commercially available sensors can currently claim.

The understanding of the methods for NO concentration quantification will also assist in the development of equations to quantify intracellular NO concentrations in vitro and extracellular NO concentrations in vivo.

## 4. Conclusions

Two methods of NO concentration quantification have been developed, both for real-time and longer time period data collection modalities. The two equations that we developed, specifically the equation derived from the difference in the initial and final signal intensity and the equation derived from the changes (slope) in fluorescence intensity over time are *x* = (*y* − 28.59)/3.73 (with *x* = NO concentration in µM and *y* = difference in fluorescence intensity) and *x* = − (*y* + 0.21)/0.42 (with *x* = NO concentration in µM and *y* = slope of signal intensity), respectively. These two methods have limits of detection of 0.1 to 10 µM (difference in signal intensity) and 0 to 10 µM (slope of signal intensity). These equations allow for the determination of NO concentration and spatial resolution when imaging, opening up possibilities that could not be previously explored via the standard detection method of an electrochemical probe.

With this work, we have improved a tool for the study of NO in living systems by finding a mathematical equation that correlates changes in SWNT fluorescence with NO concentration. We have also created a template for the development of mathematical relationships between other SWNT sensors and their analyte. As this is the first publication demonstrating the quantification of NO concentration with a SWNT sensor, we hope that our technique can be used to improve the function of other SWNT sensors too.

## Supplementary Materials

The following are available online at https://www.mdpi.com/2079-4991/11/1/243/s1, File S1: computer program to extract the average brightness for each frame of the video.

## Abbreviations

SWNT	Single-walled carbon nanotubes
NO	nitric oxide
(AT)_15_	a 30-mer of DNA consisting of adenine and thymine repeated 15 times
HRP	horseradish peroxidase
