# Supplementary material for: Quantification of Nitric Oxide Concentration Using Single-Walled Carbon Nanotube Sensors
Source: Nanomaterials (Basel). 2021 Jan 18;11(1):243. doi: 10.3390/nano11010243 (PMC7831316; doi:10.3390/nano11010243)
Supplement: Supplementary file 1 [file nanomaterials-11-00243-s001.pdf]

File S1: computer program to extract the average brightness for each frame of the video

```
function Ave_Max_h5_Videos_Multiple_Subfolders(outputFileName)

directory = dir;
for k = 3:length(directory) %For each sheep folder in the main directory,
steps into the folder and generates the data for each sheep
    currentSubfolder = directory(k).name;
    cd(currentSubfolder);
    Frame_by_Frame_Ave_Max_h5_Videos_Folder(currentSubfolder,outputFileName);
    cd('..');
end

% -----------------------------------------------------------------------------
From:
% https://www.mathworks.com/matlabcentral/answers/92449-how-can-i-delete-the-
default-sheets-sheet1-sheet2-and-sheet3-in-excel-when-i-use-xlswrite
% (To remove excess default sheets from the workbook)

sheetName = 'Sheet'; % EN: Sheet, DE: Tabelle, etc. (Lang. dependent)

% Open Excel file.
objExcel = actxserver('Excel.Application');
objExcel.Workbooks.Open(fullfile(pwd, [outputFileName '.xlsx'])); % Full path
is necessary!

% Delete sheets.
objExcel.ActiveWorkbook.Worksheets.Item([sheetName '1']).Delete;
objExcel.ActiveWorkbook.Worksheets.Item([sheetName '2']).Delete;
objExcel.ActiveWorkbook.Worksheets.Item([sheetName '3']).Delete;

% Save, close and clean up.
objExcel.ActiveWorkbook.Save;
objExcel.ActiveWorkbook.Close;
objExcel.Quit;
objExcel.delete;

function
Frame_by_Frame_Ave_Max_h5_Videos_Folder(currentSubfolder,outputFileName)

%Create a column vector populated by char values containing the names of
% all of the H5 files contained in the folder.
h5FilesDirectory = dir(fullfile(pwd,'*.h5'));
for k = 1:length(h5FilesDirectory)
    allFileNames(k,:) = {h5FilesDirectory(k).name};
end

%Process each H5 video file in the folder using a subfunction
for k = 1:length(allFileNames)
    Process_Single_h5_Video(currentSubfolder,outputFileName,allFileNames{k});
end
```

```
function Process_Single_h5_Video(currentSubfolder,outputFileName,videoName)

% Retrieve the video contained in the file in matrix form, and calculate
% the number of frames in the video (the third dimension of the matrix).
video = h5read(videoName,'/Cube/Images');
videoFramesNumber = size(video,3);

% Creates an ordered collection of images - individual frames - from the
% contents of the video.
for i = 1:videoFramesNumber
    imageCollection{i,:} = video(:,:,i);
end

% Calculates the average and the maximum pixel value for every frame
% contained in the video.
for i = 1:videoFramesNumber
    singleImage = imageCollection{i};
    averages(i,:) = mean(mean(singleImage));
    maxes(i,:) = max(max(singleImage));
end

% Creates a matrix for numbering each frame of the video
frameNumber = 1:videoFramesNumber;
frameNumber = frameNumber(:);

% Coverts each of the frame, average, and max column vectors into tabular
% form for output to Excel.
frameNumbersTable = array2table(frameNumber);
averagesTable = array2table(averages);
maxesTable = array2table(maxes);

% Prevents unecessary MATLAB warnings from being generated whenever a new
% sheet is added to the Excel file
warning('off','MATLAB:xlswrite:AddSheet');

% Combines the frame number, averages, and maxes tables into a single
% table, and outputs the results to a spreadsheet, on a new sheet labeled
% according to the name of the video file to which the data belong.
completeTable = [frameNumbersTable averagesTable maxesTable];
directory = dir('..');
writetable(completeTable,['../' outputFileName
'.xlsx'],'sheet',[currentSubfolder ' ' erase(videoName,'.h5')]);
```
